# Short and long-term 2100 MHz radiofrequency radiation causes endoplasmic reticulum stress in rat testis

**DOI:** 10.1007/s00418-024-02308-7

**Published:** 2024-07-12

**Authors:** Esma Kirimlioglu, Asli Okan Oflamaz, Enis Hidisoglu, Sukru Ozen, Piraye Yargicoglu, Necdet Demir

**Affiliations:** 1 Departments of Histology and Embryology, Faculty of Engineering, Akdeniz University, Antalya, Turkey; 2 Present Address: Departments of Biophysics, Faculty of Medicine, Faculty of Engineering, Akdeniz University, Antalya, Turkey; 3 Department of Electrical and Electronics Engineering, Faculty of Engineering, Akdeniz University, Antalya, Turkey; 4 Present Address: Faculty of Medicine, Department of Histology and Embryology, Bozok University, Yozgat, Turkey; 5 Present Address: Department of Drug Science, NIS Centre, University of Turin, Turin, Italy

**Keywords:** Male infertility, Radiofrequency radiation, Endoplasmic reticulum stress, Apoptosis, Grp78

## Abstract

Long-term radiofrequency radiation (RFR) exposure, which adversely affects organisms, deteriorates testicular functions. Misfolding or unfolding protein accumulation in the endoplasmic reticulum (ER) initiates an intracellular reaction known as ER stress (ERS), which activates the unfolded protein response (UPR) for proteostasis. Since both RFR exposure and ERS can cause male infertility, we hypothesized that RFR exposure causes ERS to adversely affect testicular functions in rats. To investigate role of ERS in mediating RFR effects on rat testis, we established five experimental groups in male rats: control, short-term 2100-megahertz (MHz) RFR (1-week), short-term sham (sham/1-week), long-term 2100-MHz RFR (10-week), and long-term sham (sham/10-week). ERS markers Grp78 and phosphorylated PERK (p-Perk) levels and ERS-related apoptosis markers Chop and caspase 12 were investigated by immunohistochemistry, immunoblotting, and quantitative real-time polymerase chain reaction (qPCR). Long-term RFR exposure increased Grp78, p-Perk, and Chop levels, while short-term RFR exposure elevated Chop and caspase 12 levels. Chop expression was not observed in spermatogonia and primary spermatocytes, which may protect spermatogonia and primary spermatocytes against RFR-induced ERS-mediated apoptosis, thereby allowing transmission of genetic material to next generations. While short and long-term RFR exposures trigger ERS and ERS-related apoptotic pathways, further functional analyses are needed to elucidate whether this RFR-induced apoptosis has long-term male infertility effects.

## Article Highlights

- Long-term radiofrequency radiation in testis triggers endoplasmic reticulum stress.
- Short-term radiofrequency radiation causes apoptosis in testis.
- Spermatogonia and spermatocytes are not affected by short-term radiofrequency radiation.

## Introduction

Radiofrequency radiation (RFR) exposure spreads rapidly through various technological devices. GSM networks operate at 900 MHz, while 2100 MHz enables higher data speeds for third-generation (3G) mobile phones. Both systems are widely used in our daily lives. RFR is suggested to affect adversely human health worldwide. Previous studies reported several adverse effects of RFR on reproductive health and infertility (Lin et al. 2017; Singh et al. 2018; Aydogan et al. 2015; Panagopoulos et al. 2010). Prolonged exposure to RFR via mobile phones increased oxidative stress and decreased gonadotropic hormone, thereby decreasing testicular function (Oyewopo et al. 2017). Increased RFR-mediated oxidative stress causes germ cell DNA damage, which alters cell cycle progression in mice, resulting in a reduced sperm count (Pandey et al. 2017). Two previous studies of liver and brain tissues in rats showed that long-term 2100 megahertz (MHz) RFR exposure caused adverse effects associated with enhanced oxidative stress, and 950 MHz RFR exposure did not induce oxidative stress (Furtado-Filho et al. 2014). Researchers have also reported that short-term 2100 MHz RFR exposure reduces oxidant levels and increases antioxidant activities, which may have protective effects on oxidant/antioxidant status (Hidisoglu et al. 2016).

Male reproductive organs, especially, are heavily exposed to RFR, since most young and middle-aged men carry their mobile phones close to their scrotal areas. A relationship between increased male infertility and RFR exposure of the testicles is reported (Agarwal et al. 2011; Karaman et al. 2014). In support this relationship, impaired expression of heat shock proteins, superoxide dismutase, peroxiredoxin-1, and misfolded proteins were detected in RFR-exposed rat testicular tissues (Sepehrimanesh et al. 2014).

The endoplasmic reticulum (ER) is an organelle with many cellular functions. In ER, Ca^2+^-dependent molecular chaperones, such as glucose-regulated protein (Grp)78, Grp94, and calreticulin, play roles for appropriate protein folding and transport. Protein homeostasis in the ER is critical in cell life. Unfolded protein response (UPR) is a signaling network that triggers ER stress (ERS), which is the process of damaged proteostasis resulting from an overload of misfolded and/or unfolded proteins in the ER (Xu et al. 2005; Liu et al. 2021; Keczan et al. 2016).

**Fig. 1:**
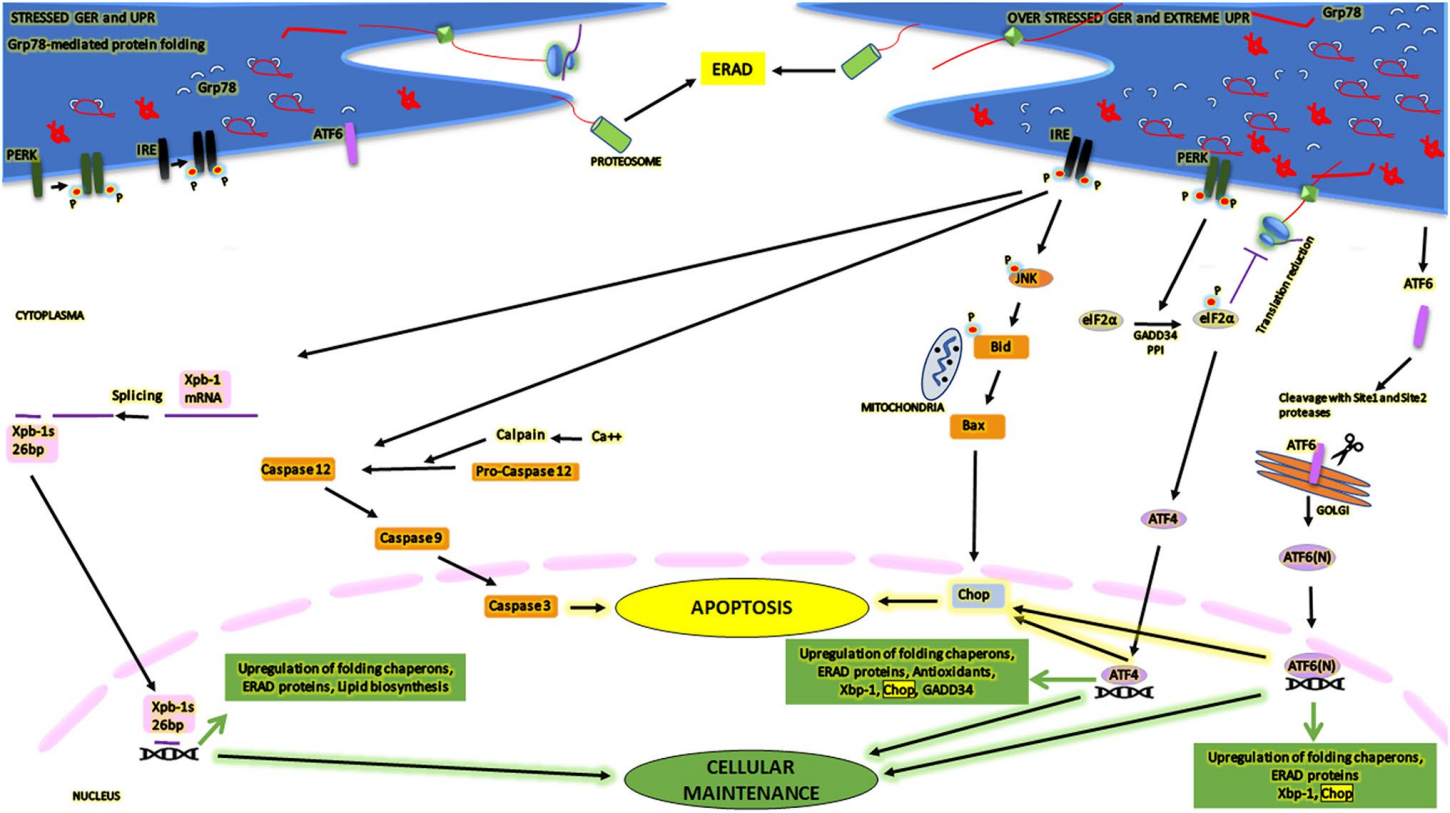
ERS and ERS-mediated apoptotic pathway. Chaperone proteins such as Grp78 initiate the UPR in the granular endoplasmic reticulum (GER) membrane. The ER molecular sensor proteins (IRE-1, PERK, and ATF-6) are inhibited by Grp78 under normal conditions but activated upon accumulation of unfolded proteins. This activation regulates protein synthesis and triggers ER-associated degradation (ERAD) or autophagy. IRE-1, when activated, triggers the splicing of Xbp-1 mRNA, regulating stress response proteins. PERK phosphorylates eIF2α, decreasing global protein synthesis but upregulating selective mRNA translation. ATF6 translocates to the Golgi and becomes a transcription factor, regulating UPR target genes. Prolonged UPR activation can lead to cell death via caspase 12, Chop, and IRE1–JNK pathways. ERS-induced apoptosis involves Chop, Bcl-2, JNK, and caspases. Caspase 12 activation is linked to ER stress rather than mitochondrial signals

The ER membrane contains UPR sensor proteins called inositol-requiring enzyme (IRE)-1, protein kinase RNA-like endoplasmic reticulum kinase (PERK), and activating transcription factor (ATF)-6 transmembrane proteins. Under physiologic condition, these UPR sensors bind to the chaperone protein Grp78, which prevents activation of these sensors, thereby inhibiting UPR signaling. Accumulation of the unfolded proteins in the ER lumen dissociates Grp78 from these UPR sensor proteins. The release of Grp78 leads to the homeostasis of protein synthesis via autophagy or activation of ER-associated degradation (ERAD) processes to clean unfolded and misfolded proteins. After Grp78 dissociation, IRE1 is oligomerized and phosphorylated, and its activated endoribonuclease domain cleaved X-box-binding protein 1 (Xbp-1) mRNA forms a splice form of the Xbp-1 mRNA (sXbp-1), which is then imported to the nucleus to induce expressions of chaperone proteins Grp78 and Grp94. Then, if misfolded proteins still increase, the ERAD mechanism is activated to degrade them (Chen and Brandizzi 2013; Carlesso et al. 2019). Besides ERAD degradation, both PERK and IRE1 can trigger autophagy via ERS. Phosphorylated PERK (p-Perk) after activation of PERK phosphorylates the elongation induction factor 2α (eIF2α) and down-regulates global protein synthesis. eIF2α upregulates translational inhibition of several mRNAs while inducing Chop expression via ATF4. Moreover, ATF6 translocates to the Golgi apparatus during ERS and is cleaved by site-1 protease (SP1) and site-2 protease (SP2). The N-terminal part of cleaved form becomes an active transcription factor that regulates the expression of Chop, Xbp1, and chaperones (Fulda et al. 2010). If the unfolded protein levels exceed the ER folding capacity, cells undergo ERS-induced apoptosis mediated by caspase 12, Chop, and IRE1–JNK pathways. Prolonged ERS causes the IRE1–TRAF2 complex- and calpain-mediated release of the proapoptotic caspase 12. Conditions inducing ERS and disrupting calcium balance but not mitochondria-mediated apoptotic signals, triggering activation of caspase 12, which subsequently activates the caspase 9-mediated caspase 3 pathway, resulting in ERS-mediated apoptosis (Nakagawa et al. 2000; Rao et al. 2002; Lee et al. 2005) (Fig. 1).

Apoptosis is detected in male germ cells exposed to low-dose radiation (Hamer et al. 2001). However, apoptosis as a physiologic mechanism also contributes to the clearance of overproduced, damaged, or genetically abnormal germ cells during spermatogenesis (Koji and Hishikawa 2003; Sinha Hikim et al. 2003). Studies have shown that exposure to 950 MHz RFR does not cause oxidative stress (Furtado-Filho et al. 2014). While long-term 2100 MHz RFR exposure enhances oxidative stress in brain, short-term 2100 MHz RFR exposure protects tissue by enhancing antioxidative status (Hidisoglu et al. 2016). Several studies (Lin et al. 2017; Oyewopo et al. 2017; Pandey et al. 2017) emphasize that RFR causes structural and cellular changes that affect male fertility. Similarly, ERS is also associated with male and female infertility as well as preimplantation embryo development (Guzel et al. 2017; Basar et al. 2014). This leads us to hypothesize that short- and long-term exposures of rat testis tissue to 2100 MHz RFR adversely affect testicular functions by stimulating ERS signaling. Therefore, we investigate the impacts of short- and long-term 2100 MHz RFR exposure on activation of ERS proteins and ERS-mediated apoptotic cascades in rat testes.

## Materials and methods

### Ethical approval and animal care

Akdeniz University Experimental Animals local ethics committee approved this protocol with the decision numbered B.30.2.AKD.0.05.07.00/30. Experimental protocols applied for rats were determined in accordance with the Akdeniz University Faculty of Medicine, Animal Care and Use Committee standards. Male albino Wistar rats, 3 months old, weighing 200–250 g, were housed in stainless steel cages (four rats per cage) and given food and water ad libitum. Animals were maintained on 12-h light–dark cycles and at a constant temperature of 23 ± 1 °C.

### Study design and radio frequency radiation application

Rats were randomly divided into five groups (*n* = 10 per group): group 1: Control; group 2: Short-term 2100 MHz RFR (1 week); group 3: Short-term sham (sham, 1 week); group 4: Long-term 2100 MHz RFR (10 week); group 5: Long-term sham (sham, 10 week). During the experiment, each rat was placed in perforated plexiglass tubes to facilitate breathing and reduce the increase in body temperature. The 1-week and 10-week groups were exposed to 2100 MHz RFR emitted from the generator for 2 h a day for 1 and 10 weeks, respectively. Sham rats were housed under the same conditions for an equal amount of time in separate rooms without exposure to RFR. All researchers participated blind to data collection, analysis, and experimental group formation.

A radio frequency (RF) generator (UMTS Simulator 2.1 GHz; Everest Company, Adapazarı, Turkey), which produces 2100 MHz RFR, was used to represent the exposure of the Universal Mobile Telecommunications System (UMTS). The output power of the generator was fixed at 1.5 W during exposure. The modulation frequency was 217 Hz, the pulse width was 0.577 ms, and the power range of the generator was 0–2 W. During exposure, rats were placed in specially designed plexiglass tubes with air holes to assist breathing and prevent the rise in body temperature.

**Fig. 2:**
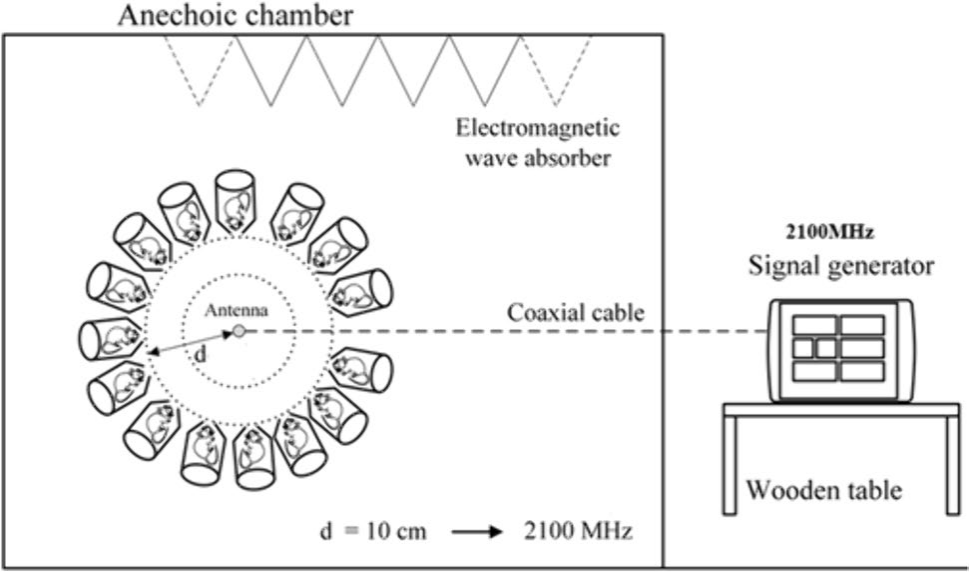
Setup of the electromagnetic exposure system

Rats in plexiglass tubes were placed radially at equal distances around the antenna (rat noses were 10 cm away from the antenna) (Fig. 2). The carousel setup procedure in this study was applied as described previously (Burkhardt et al. 1997; Fritze et al. 1997; Schonborn et al. 2004). The tubes restrained the movement of the rats to such an extent as to follow well-defined exposure conditions, yet without immobilizing them. RFR application was held in a shielded room to protect the rats from the effects of other electrical sources. For the frequency, the output powers of the source were selected by considering the power values emitted by the mobile phones (the output power was adjusted to 1.5 W during the exposure), and the electric field values were measured. During the “signal on” experiment, the measured electric field strengths over the rat’s head positioned 10 cm away from the antenna were 35.2 V/m for 2.1 GHz.

Electric field strengths were measured by EMR300 (Narda, Germany) with a suitable probe in the experiment. The electric field background level of the shielded room was between 0.02 and 0.2 V/m. Also, the background magnetic field was measured between 0.01 and 0.03 µT by Hioki 3470 Magnetic Field Hitester (Hioki E. E. Corp., Japan) with an appropriate probe. Dosimetry simulations were carried out using a finite integration technique (FIT) based commercial software, CST Microwave Studio (3DEXPERIENCE®, Dassault Systems, Hamburg). The FIT was introduced by Weiland (Weiland 1977). Although the gridding can be applied as a finite difference time domain (FDTD) method, the FIT uses the integral form of Maxwell’s equations (Razi-Kazemi 2018). In the present study, the rat model used in simulations consisted of voxels with a resolution of 1.827 × 1.827 × 2.015 mm^3^. The interaction between the incident electromagnetic wave and the biological tissue is explained by electrical properties that can be obtained by the dielectric properties of the tissue of interest (Gabriel et al. 1996; Abdilla et al. 2013). In this study, each tissue of the simulated rats had electrical properties at the operating frequencies. The average whole-body SAR value at 2100 MHz was 0.16 W/kg. The SAR value for the testes was an average of 0.0347 W/kg. Before and after all experimental sessions, the body temperatures of rats were monitored by rectal measurements. The RFR exposure did not cause elevation in rectal temperature.

### Collection and preparation of biological samples

After RFR administration, rats were euthanized by cervical dislocation under ether anesthesia, and the testicles were removed. After washing with 0.9% heparinized saline, one testis of each rat was placed in bouin fixative and kept at +4 °C for 12 h, and a tissue follow-up procedure was performed. The other testis of the rats was divided transversally into two and placed in liquid nitrogen to use one half in quantitative real-time polymerase chain reaction (qPCR) and the other half in immunoblot analysis.

### Tissue processing

Tissues taken into bouin fixative were trimmed during the fixation process. Following the fixation, testis samples were passed through ascending alcohol series as described (Okan et al. 2024), they were washed in tap water overnight. After the tissues became completely transparent in xylol, paraffinization was performed, and the tissues were embedded in paraffin. Then, 5-µm sections from paraffin blocks were taken on Superfrost slides using a rotary microtome (Leica, Nussloch, Germany).

### Preparation of protein lysate

Testicular tissues removed from the nitrogen tank were placed in sterile +4 °C cold glass and mechanically dissected with a scalpel. Next, they were taken in a lysis buffer (0.1 M Tris pH 7.4, Sodium-orthovanadate, SDS) supplemented with a competent protease inhibitor cocktail tablet (P8340; Sigma Aldrich), and tissues were homogenized using homogenizer (Bandelin electronics HD 2070) for 1 min and sonicated using a sonicator (Bandelin sonopuls GM 2070) for 6 s. The samples were then centrifuged for 10 min at 25200*g* at +4 °C. Finally, the supernatant was collected for immunoblotting analysis.

### Isolation of total RNA and preparation of cDNA

After the testicular tissues were removed from the nitrogen tank, they were placed in +4 °C cold glass under sterile conditions and quickly mechanically dissected using a scalpel. Testis tissues were then placed in Trizol reagent (Life Technologies, Darmstadt, Germany) for total RNA isolation. The RNA concentration was calculated by measuring absorbance at 260 and 280 nm using the EPOCH nanodrop system. After applying DNase I (Ambion, Austin, TX) to 10 μg RNA to eliminate genomic DNA contamination, complementary DNA (cDNA) was generated using the RETROScript kit (Ambion, Austin, TX).

### Immunohistochemistry

The streptavidin–biotin peroxidase method was used as previously described (Okan et al. 2021). Briefly, paraffin sections (5 µm) were deparaffinized and then rehydrated. The sections were boiled with sodium citrate buffer pH 6.0 for heat-mediated antigen retrieval in a microwave. Then, the slides were placed in 3% hydrogen peroxide (H_2_O_2_) (Sigma-Aldrich) for blocking of endogenous peroxidase activity. Thereafter, ultra V block (#TA-125-UB; Thermo Scientific/Lab Vision) solution was applied for protein blocking. Sections were then incubated with primary antibodies (respectively; Grp78, ab21685 Abcam,1:600; p-Perk, bs-3330R Bioss, 1:100; caspase 12, PA5-19,963 Thermo Fisher Scientific, 2 μg/ml; Chop (Ddit3), ab179823 Abcam, 1:50) at +4 °C overnight. To test secondary-dependent specificity, primer-free antibody diluent was used in negative control staining After several rinses in PBS, secondary antibody (BA-1000 Vector Laboratories, 1:400) was applied for 1 h and followed by several rinses in PBS; the slides were then incubated in streptavidin–peroxidase complex (TS-125-HR, THERMO) for 30 min at room temperature. After several washes in PBS, 3,3′-diaminobenzidine (DAB) substrate (TA-012-HDC- TA-125-HDS) was used to visualize the peroxidase reaction. Counterstaining of sections with Mayer’s hematoxylin solution (#1.09249.1000; Merck), was followed by dehydration and mounting with Entellan (#1.07961.0100; Merck). Images were captured using ZEN 2.5 lite program in a bright field microscopy (Zeiss-Primostar), and expression levels of each protein in all groups were analyzed using ImageJ software.

### Immunoblotting

The sodium dodecyl sulfate–polyacrylamide gel prepared in this study was 15% for Chop and 10% for other proteins. After electrophoresis, proteins were electrotransferred to the nitrocellulose membrane. The target protein is labeled with the following antibodies as follows: rabbit anti-Grp78 antibody (ab21685 Abcam,1: 500 in 5% skim milk powder-TBS-t), rabbit polyclonal Chop (Ddit3) antibody (ab179823 Abcam, 1: 250 in TBS-t), rabbit anti-p-Perk antibody (bs-3330R Bioss, 1: 250 in TBS-t), rabbit polyclonal anti-caspase 12 antibody (PA5-19963 Thermo Fischer Scientific, 1 μg/ml in TBS-t), and beta-actin (4970 Cell Signaling Technology, 1: 1000 in 5% skim milk powder-TBS-t). Immunodetection was performed using HRP-labeled goat anti-rabbit secondary antibody (PI-1000 Vector Laboratories), and then protein levels were detected using an enhancer chemiluminescent (ECL) substrate (THERMO-32106).

### Quantitative real-time PCR (qPCR)

PCR reactions (25 μl) mix contained 12.5 μl 2 × SYBR green superMix (Qiagen), 0.5 μl of each specific primer (10 μM), 10 ng first-strand cDNA, and nuclease-free water. Gene-specific primers for *Hspa5 (Grp78)* and *Ddit3 (Chop*) are given in Table 1. The qPCR reactions were performed on a Rotor-Gene (Corbett Research). Beta-actin (*β-actin*) was used as an internal control housekeeping gene to normalize the expression of target genes.

**Table 1:** List of primers used in qPCR analysis

*Hspa5* forward primer	5′-CCACCGTAACAATCAAGGTC-3′
*Hspa5* reverse primer	5′-CACGTGAGCAACTGCTAATG-3′
*Dditt* forward primer	5′-GAAATCGAGCGCCTGACCAG-3′
*Dditt* reverse primer	5′-GGAGGTGATGCCAACAGTTCA-3′
*β-actin* forward primer	5′-TACAGCTTCACCACCACAGC-3′
*β-actin* reverse primer	5′-AAGGAAGGCTGGAAAAGAGC-3′

The qPCR program included an initial denaturation at 95 °C for 5 min followed by 30 cycles set at 92 °C for 30 s for denaturation, 65 °C for 20 s for annealing, and 72 °C for 60 s for elongation, and the subsequent 35 cycles were set at as 92 °C for 20 s, 55 °C for 15 s, and 72 °C for 60 s. A dissociation step cycle (55 °C for 10 s and then 0.5 °C for 10 s until 95 °C) was added for melting curve analysis. Three technical replicates and two biological replicates were analyzed for each specimen. As a result of the analysis of the samples, qPCR was created with reference DNA in the standard range. *Hspa5*, *Ddit3*, and *β-actin* expression levels were quantitatively determined in the threshold cycle and other cycles obtained by reading on the instrument (Rotor-Gene-QIAGEN) and using the software program. Delta Ct (ΔCt) indicates the difference between the expression levels of the target gene and the housekeeping gene. ΔCt values were determined by subtracting the Ct value given by the endogenous control from the Ct value of the tested gene separately for each sample. The relative mRNA levels were calculated using 2^−ΔΔCT^values.

### Statistical analysis

The normalization test was performed using the Kolmogorov–Smirnov test. A one-way analysis of variance (ANOVA) and post hoc Tukey test was applied using GraphPad (Prisms10) program to determine statistical significance between the groups. Values with *P* < 0.05 were considered statistically significant. Results are presented as mean ± standard error of the mean (SEM).

## Results

### Long-term RFR exposure triggers ERS in rat testes

**Fig. 3:**
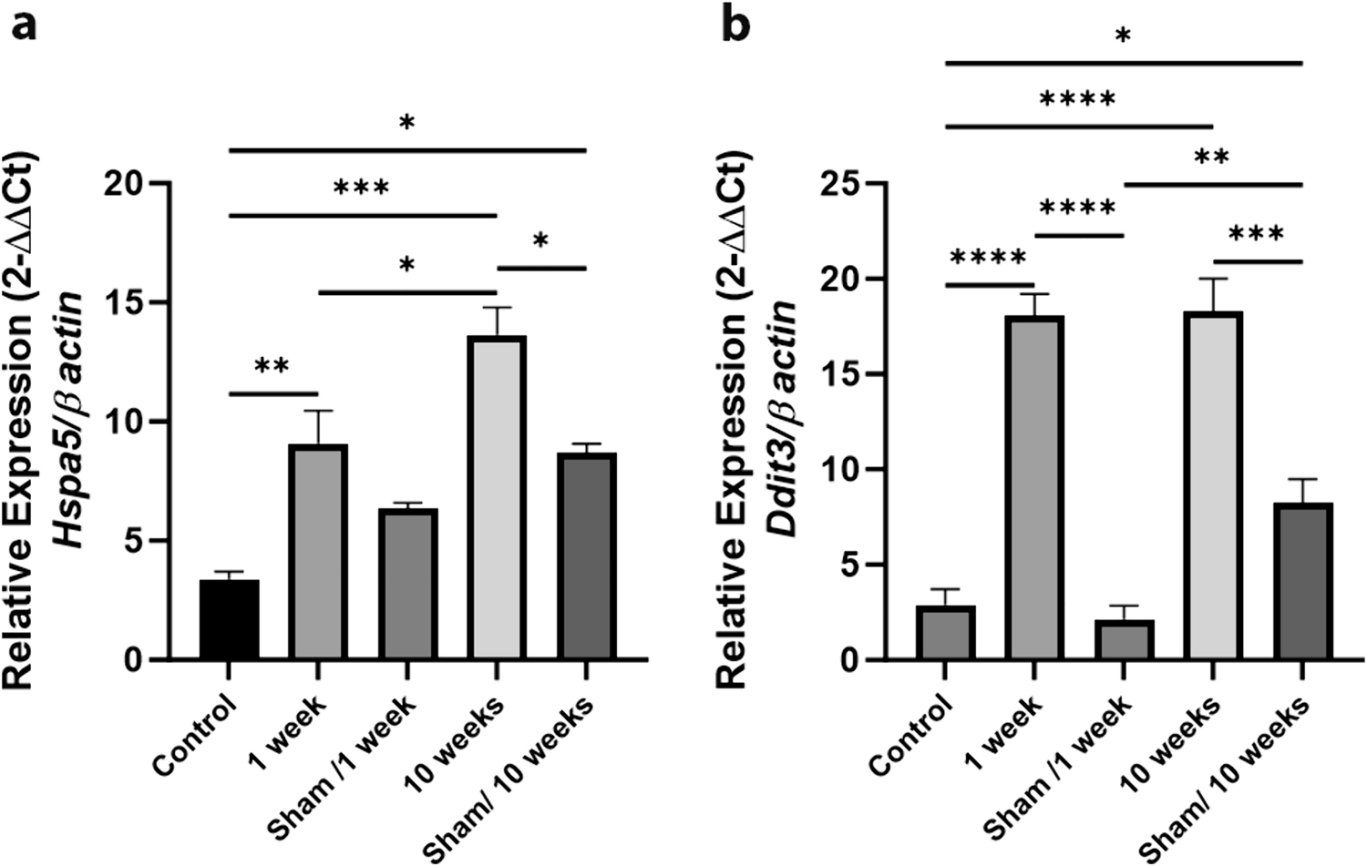
ERS-related protein gene expression increased dependent on the exposure time of RFR. The relative mRNA expression of *Hspa5* (**a**) and *Ddit3* (**b**) was evaluated by determining the ratio of the expression of the target mRNA to the *β-actin* mRNA. Statistical analyses were performed using a one-way analysis of variance (ANOVA) followed by a post hoc Tukey’s multiple comparisons tests; data are expressed as the mean ± SEM (bars represent mean ± SEM, *P < 0.05; **P < 0.01; ***P < 0.001; **** *P* < 0.0001)

**Fig. 4:**
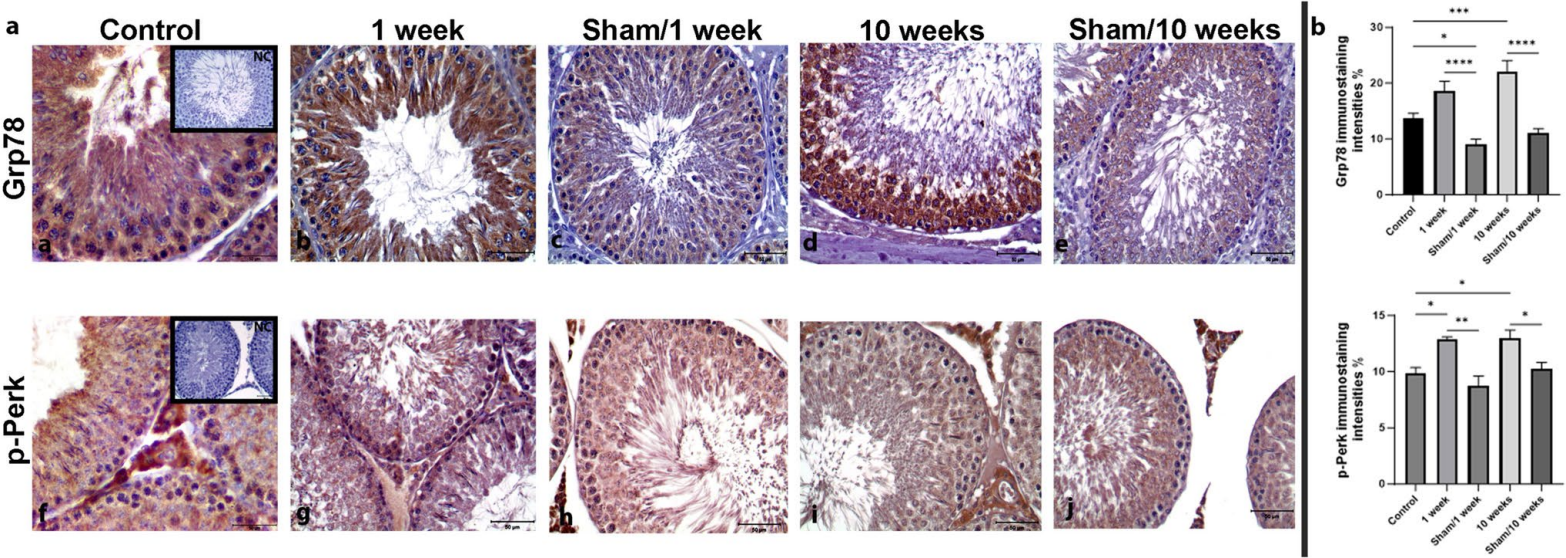
Short and long-term RFR exposure increased Grp78 and p-Perk expression levels in rat testicular tissues. **a** The analyses of ERS-associated proteins Grp78, and p-Perk by immunohistochemistry. Increased expression of Grp78 (**a**–**e**) and p-Perk (**f**–**j**) were observed in all the spermatogenesis stages in 1-week and 10-week 2100 MHz RFR groups. p-Perk expression was observed in the cytoplasm of primary spermatocytes for the 1-week RFR group and in elongated spermatocytes for the 10-week RFR group in the seminiferous tubule. NC, negative control has no staining. **b** Histograms represent the intensity values in percent of immunostaining obtained using ImageJ software. Statistical analyses were performed using one-way analysis of variance (ANOVA) followed by a post hoc Tukey’s multiple comparisons test; data are expressed as the mean ± SEM (*P < 0.05; **P < 0.01; ***P < 0.001; ****P < 0.0001. Scale bars, 50 μM. Objective, 40×)

**Fig. 5:**
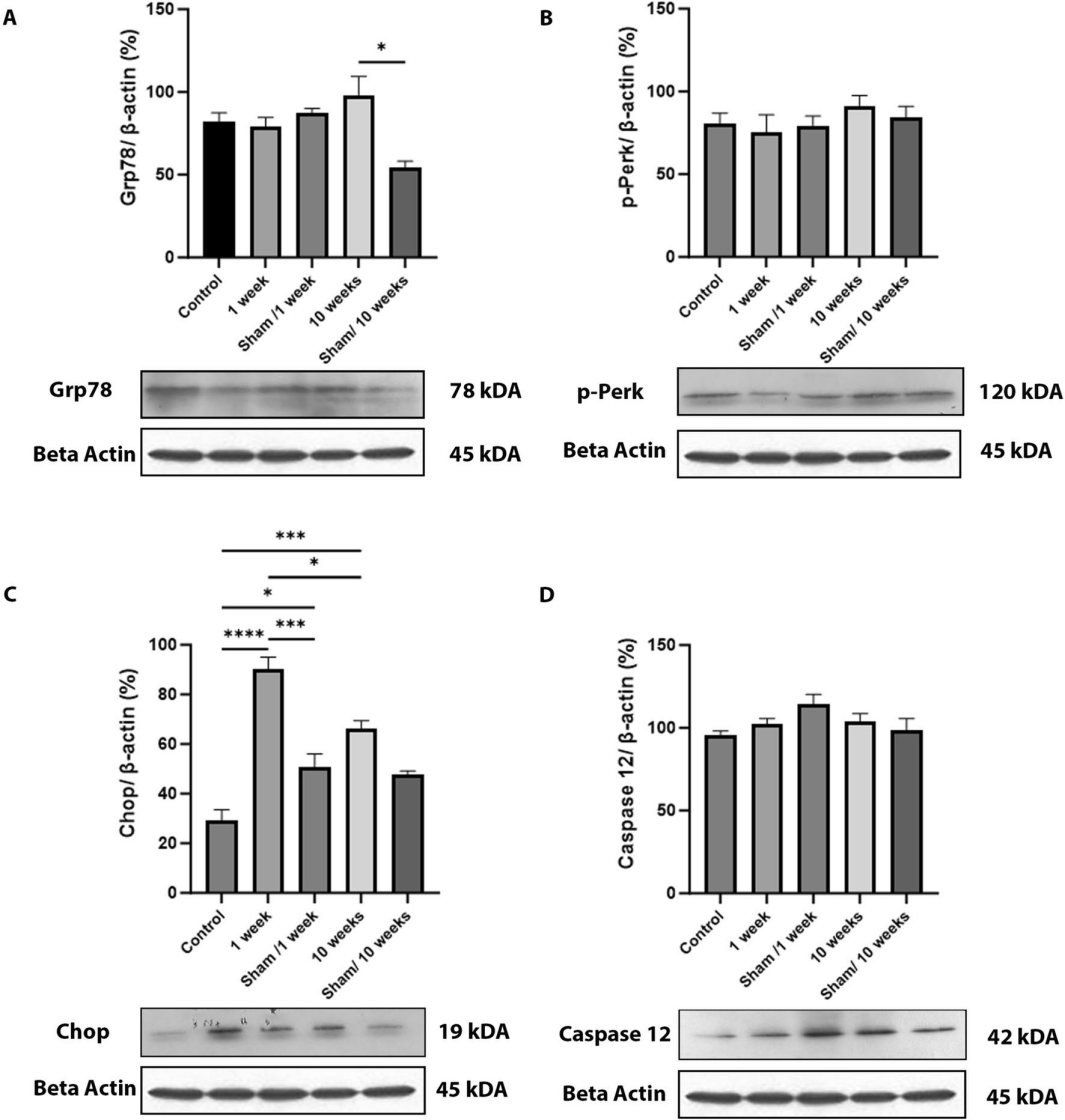
RFR exposure induced Chop, the ERS-mediated apoptotic marker. The detection of the endogenous expression profiles and quantification results of Grp78 (**A**), p-Perk (**B**), Chop (**C**), and caspase 12 (**D**) in testicular tissues from the control, 1-week and 10-week exposed 2100 MHz RFR groups, and sham/1-week and sham/10-week groups by immunoblotting analysis. The protein expression of Grp78 increased in 10-week 2100 MHz RFR group compared with sham/10-week groups. The protein expression of Chop increased in 1-week 2100 MHz RFR group compared with the other experimental groups. The protein expression of p-Perk and caspase 12 were not significantly different among groups. β-actin normalized the relative densities of each protein expression. Statistical analyses were performed using one-way analysis of variance (ANOVA) followed by post hoc Tukey’s multiple comparisons test); data are expressed as the mean ± SEM (*P < 0.05; ***P < 0.001; ****P < 0.0001)

To test the RFR effect on initiation of ERS in rat testis tissue, we evaluated the expression levels of mRNA and protein of Grp78, the primary chaperone of ERS cascade. The mRNA level of *Hspa5* increased in the rat testicular tissues of the 1-week (*P* < 0.01) and 10-week (*P* < 0.001) RFR groups compared with controls (Fig. 3). Immunohistochemical analysis revealed that Grp78 protein expression was increased in both 1-week RFR groups (*P* < 0.0001) and 10-week RFR groups (*P* < 0.0001) compared with their sham groups. Localization of the Grp78 protein expression was cytoplasmic in all spermatogenic series of seminiferous tubules (Fig. 4). The results of the immunoblotting analysis confirmed an increase in Grp78 protein in the 10-week RFR groups as total protein amount of the rat testis compared with the 10-week RFR sham group (*P* < 0.05) (Fig. 5).

As noted in the introduction, the increased phosphorylated level of Perk (p-Perk) is another indicator of ERS. Elevated p-Perk results in further increase Grp78 levels. In immunohistochemical assessment, the amounts of p-Perk protein were increased in both 1-week (*P* < 0.01) and 10-week (*P* < 0.05) RFR groups compared with their sham groups. In the seminiferous tubules, expression of p-Perk was observed in the cytoplasm of primary spermatocytes for the 1-week RFR group and in elongated spermatocytes for the 10-week RFR group (Fig. 4). In immunoblotting analysis, no significant difference was detected for total protein amount of p-Perk across all groups (Fig. 5).

### 1-week RFR exposure stimulates the ERS-mediated apoptotic pathway in rat testis but not in spermatogonia and spermatocytes

**Fig. 6:**
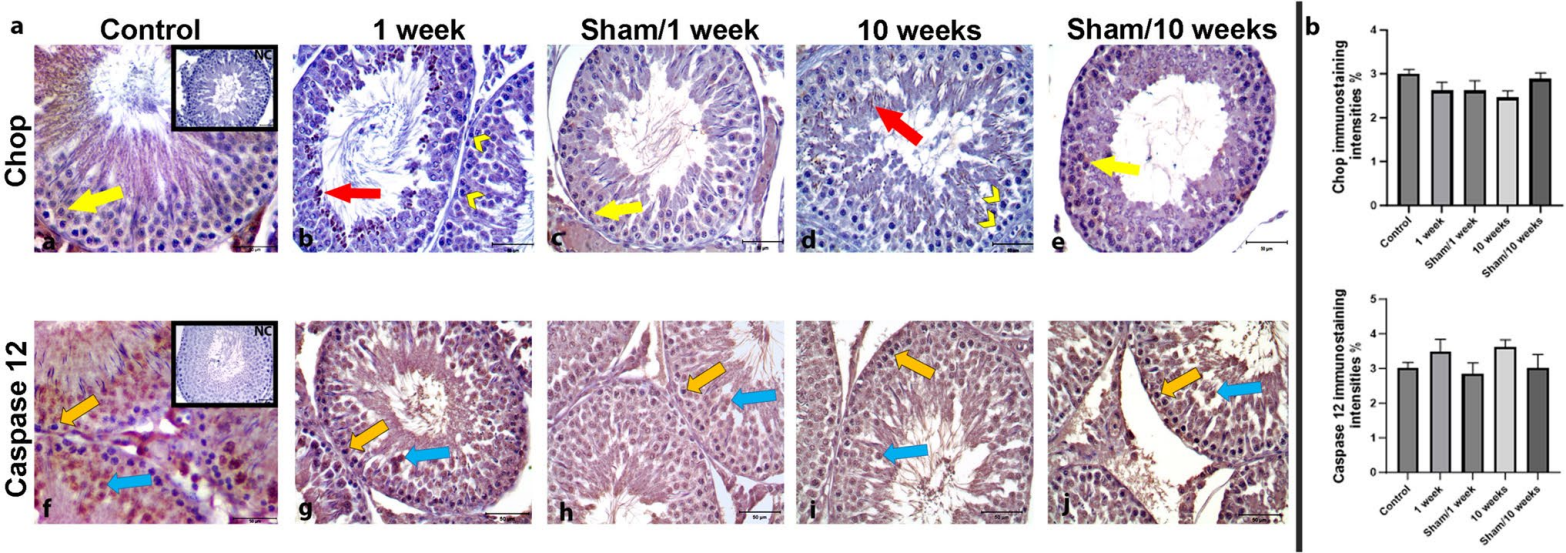
1-week RFR exposure did not induce Chop expression to spermatogonia and primary spermatocytes. **a** The analyses of ERS-associated proteins Chop (**a**–**e**) and caspase 12 (**f**–**j**) by immunohistochemistry. Chop expression was cytoplasmic in all the spermatogenesis stages of control and sham treatment groups (yellow arrows). On the other hand, nuclear Chop (red arrows) expression was observed in spermatids in seminiferous tubules of 1-week and 10-week exposed to 2100 MHz RFR groups, respectively. Chop expression was not observed in some spermatogonia and primary spermatocytes in the 1-week RFR group (Fig. 6, yellow arrowhead). Both cytoplasmic (orange arrow) and nuclear (blue arrow) expression of caspase 12 was not significantly different among experimental groups. NC, negative control has no staining. **b** Histograms represent the intensity values in percent of immunostaining obtained using ImageJ software. Statistical analyses were performed using one-way analysis of variance (ANOVA) followed by post hoc Tukey’s multiple comparisons test; data are expressed as the mean ± SEM (Scale bars, 50 μM. Objective, 40×)

Chop and caspase 12 activate the ERS-mediated apoptotic pathways. Both mRNA (*P* < 0.0001) and protein (*P* < 0.0001) levels of Chop increased in both 1-week and 10-week RFR groups compared with both sham and control groups, as determined by qPCR (Fig. 3) and immunoblotting (Fig. 5). Caspase 12 protein expression on immunoblotting was similar in all group (Fig. 5). However, neither Chop nor caspase 12 levels displayed any difference in cell types of rat seminiferous tubes among the groups (Fig. 6). Moreover, no Chop immunoreactivity is observed in spermatogonia, while weak Chop staining is seen in primary spermatocytes (Fig. 6). On the other hand, nuclear Chop expression was detected in spermatids in seminiferous tubules of 1-week and 10-week rat groups exposed to 2100 MHz RFR (Fig. 6).

## Discussion

Deteriorating environmental conditions due to developing technology cause cellular stress including oxidative stress and/or ERS. Evolutionarily, cells develop various mechanisms to deal with these stress conditions over time and to reestablish intracellular homeostasis. Acute or chronic stress conditions may cause protein folding errors or prevent protein folding, resulting in accumulation of misfolded or unfolded proteins in the ER lumen. Excessive folding capacity of the ER activates three molecular sensor proteins in the ER membrane (IRE1, PERK, and ATF6) to initiate the UPR response. Subsequently, transcriptional, and translational adaptation mechanisms are processed. Thus, to reduce the load on the ER lumen, global protein synthesis is inhibited while the expression of chaperones increases to compensate protein folding together with degradation of misfolded or unfolded proteins. Despite all these mechanisms, apoptotic pathways can be activated if a cell cannot overcome ERS due to an overloaded levels of misfolded or unfolded proteins. As the most important marker of ERS, Grp78 chaperone increases to adjust protein folding under ERS. Moreover, the increased level of p-Perk activates (phosphorylates) eIF2α, thereby inhibiting global translation. If stress increases further and prolongs, ATF4-mediated Chop and caspase apoptotic pathways are activated (Cao and Kaufman 2014) (Fig. 1).

Our total Grp78 levels were significantly elevated in rat testicular tissue from the 10-week RFR group compared with the control. In support, Chop mRNA and protein expression was also increased in the 1-week and 10-week RFR group relative to other groups. The increase of both Grp78 and Chop in 1-week and 10-week groups is an important indicator of the presence of testicular ERS induced by RFR exposure, suggesting that cell hemostasis may be impaired by RFR-mediated excessive ERS, leading to disruption of spermatogenesis. Noticeable increase in Chop expression in spermatids in 1-week and 10-week group support this premise. Our results are supported by a previous study reporting that long-term exposure to 2100 MHz RFR also induces oxidative stress, which is a known ERS inducer (Hidisoglu et al. 2016).

One study suggested that IRE1 is a positive regulator of IME1 (meiosis inducer), a meiosis stimulatory regulatory gene in *Saccharomyces cerevisiae* (Schroder et al. 2000). One study revealed no significant change in cell proliferation after short-term exposure to RFR (Xu et al. 2017). Our results show that the ERS-mediated apoptotic markers Chop are increased in both short and long-term 2100 MHz RFR exposure but not in spermatogonia and primary spermatocytes in the seminiferous tubules. According to results from these previous studies, IRE1 contributes to induction of meiosis, whereas Chop contributes to ERS-mediated apoptosis. Prolonged ER stress leads to an increase in Chop expression, which in turn promotes apoptosis. Therefore, reducing Chop expression can help mitigate some of the negative effects of prolonged ER stress by decreasing proapoptotic signaling. Achieving proteostasis after endoplasmic reticulum stress involves reducing the expression of the Chop protein. Studies have shown that inhibiting Chop can alleviate some of the detrimental effects of proteasome inhibition by reducing apoptosis, suggesting a protective role when Chop activity is reduced under conditions of sustained ER stress (Hetz et al. 2015). It is possible to establish proteostasis following ERS stress activated by 2100 MHz RFR. This suggests that the lack of Chop expression in spermatogonia and primary spermatocytes may be related to their natural resistance to apoptosis induced by ERS resulting from 2100 MHz RFR. While we observed that Chop expression was increased throughout testicular tissue following both short and long-term 2100 MHz RFR exposure, its absence in spermatogonia and primary spermatocytes suggests a protective mechanism in spermatogonia and primary spermatocytes, enabling these cells to be resistant to ERS-mediated apoptosis and maintain their meiotic division to properly transmit genetic material to future generations.

## Conclusions

To our knowledge, this is the first study showing activation of ERS in testicular tissues of rats exposed to short and long RFR. Current study also revealed that short and long 2100 MHz RFR application activates the protein folding response of the rat testis as well as ERS-mediated apoptosis. However, the lack of Chop expression in the spermatogonia and primary spermatocytes in the adluminal compartment, which is the lower layer of the blood-testis barrier, may be related to a protective mechanism pertaining to both the preservation of this cell pool to transfer genetic material to future generations.

## Data Availability

Data are available on request from the authors.

## Abbreviations

ATF: Activating transcription factor
BCL2: B cell lymphoma2
JNK: C-Jun N-terminal kinase
eIF2α: Elongation induction factor 2α
ER: Endoplasmic reticulum
ERS: Endoplasmic reticulum stress
ERAD: ER-associated degradation
Grp: Glucose regulated protein
H_2_O_2_: Hydrogen peroxide
IME_1_: Inducer of meiosis
IRE: Inositol-requiring enzyme
PERK: Protein kinase RNA-like endoplasmic reticulum kinase
p-Perk: Phosphorylated PERK
RFR: Radiofrequency radiation
SP1: Site-1 protease
SP-2: Site-2 protease
UMTS: Telecommunications system
3G: Third-generation
MHz: Megahertz
UPR: Unfolded protein response
qPCR: Quantitative real-time polymerase chain reaction
*Hspa5*: Grp78 gene name
*Ddit3*: Chop gene name
